# Presence, removal, and risks of psychopharmaceuticals in wastewater streams

**DOI:** 10.1093/etojnl/vgae042

**Published:** 2025-01-06

**Authors:** Charlie J E Davey, Anne Kiki Hartelust, Rick Helmus, Antonia Praetorius, Annemarie P van Wezel, Thomas L ter Laak

**Affiliations:** Institute for Biodiversity and Ecosystem Dynamics, University of Amsterdam, Amsterdam, the Netherlands; Institute for Biodiversity and Ecosystem Dynamics, University of Amsterdam, Amsterdam, the Netherlands; Institute for Biodiversity and Ecosystem Dynamics, University of Amsterdam, Amsterdam, the Netherlands; Institute for Biodiversity and Ecosystem Dynamics, University of Amsterdam, Amsterdam, the Netherlands; Institute for Biodiversity and Ecosystem Dynamics, University of Amsterdam, Amsterdam, the Netherlands; Institute for Biodiversity and Ecosystem Dynamics, University of Amsterdam, Amsterdam, the Netherlands; KWR Water Research Institute, Nieuwegein, the Netherlands

**Keywords:** psychopharmaceuticals, wastewater, wastewater treatment plant, removal, risk

## Abstract

Psychopharmaceuticals are used to treat psychological disorders and other conditions relating to the nervous system and are known to affect nontarget organisms at low concentrations. Their occurrence in the water cycle remains an understudied topic, with data lacking for many compounds, and risks not accounted for in removal targets. Therefore, this study aimed to provide insights into the presence, removal, and risks of psychopharmaceuticals in wastewater. Furthermore, the use of risk assessment in the context of proposed legislation is discussed. Thirty highly used psychopharmaceuticals were studied during 1 week in the wastewater of the Amsterdam West Wastewater Treatment Plant (WWTP) using solid phase extraction and ultra high performance liquid chromatography–quadrupole time of flight–high resolution mass spectrometry. Twenty target compounds were detected in the influent (17 ng–99 µg/L) and 16 in the effluent (34 ng/L–17 µg/L). Removal efficiencies during treatment ranged from 24% to >99%. Paracetamol, amphetamine, fluoxetine, levetiracetam, phenacetin, and sertraline demonstrated almost complete removal, whereas tramadol, lidocaine, lamotrigine, fluvoxamine, and carbamazepine had removals below 50%, with lidocaine demonstrating the lowest removal (24%). Utilizing existing ecotoxicity data, a preliminary risk assessment was performed to contextualize the calculated removal efficiencies. Here, sertraline and ibuprofen still demonstrated a potential risk, despite high removal efficiencies of both compounds. This study highlights that wastewater contains abundant numbers and ecotoxicologically relevant concentrations of psychopharmaceuticals that are insufficiently removed by the WWTP. The implementation of risk-based removal targets in legislation is discussed to facilitate the reduction in emissions of psychopharmaceuticals, for example, by adequate WWTP upgrades with advanced treatments to ensure a toxic-free environment.

## Graphical abstract

**Figure:**
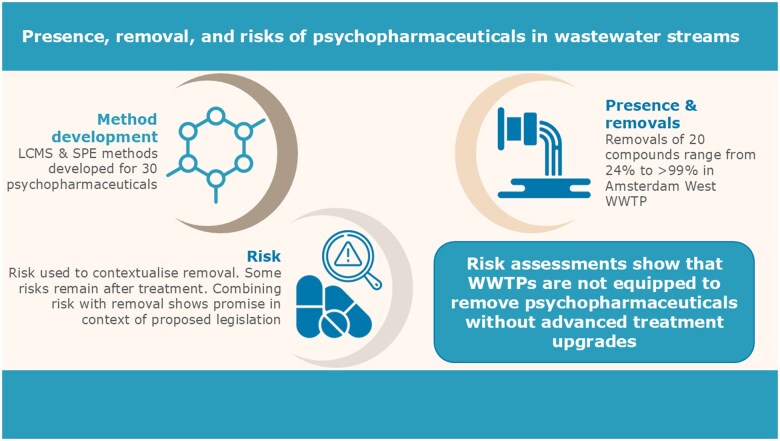

## Introduction

Psychopharmaceuticals are a subclass of pharmaceuticals designed to treat medical conditions relating to the nervous system. They are classified by the World Health Organisation as ATC-N (anatomical therapeutic class–nervous system, (World Health Organisation Collaborating Centre for Drug Statistics Methodology, 2018) and are a vital tool in modern medicine, with a growing population that uses them on a regular basis (European Medicines Agency, 2021). The growth in their use is fueled by several factors, such as growing and aging populations, a loss of social stigma of the underlying diseases (e.g., depression), and a growing number of psychopharmaceuticals entering global markets (Bean et al., 2018; Gao et al., 2013; Massey et al., 2018; Read et al., 2014; World Health Organization, 2011). In the Netherlands alone, 2.9 million people, corresponding to 17% of the population, were prescribed psychopharmaceuticals in 2021 (Zorginstituut Nederland, 2022).

After use, psychopharmaceuticals are excreted by the human body, as parent compounds and/or transformation products, and enter the sewage system. These compounds are often polar and persistent and therefore are not removed effectively by wastewater treatment plants (WWTPs). Thus, WWTPs are a continuous source of psychopharmaceuticals into the aquatic environment (Cunha et al., 2019, 2017; Delli Compagni et al., 2020; Gago-Ferrero et al., 2019; Wang et al., 2020). Psychopharmaceuticals have been shown to have adverse ecotoxicological effects on many aquatic species due to similar neurochemical architecture shared between humans and other nontarget species (Edsinger and Dölen, 2018; Liebeskind et al., 2017). Psychopharmaceuticals can alter the behavior of nontarget organisms, leading to changes in feeding, social interactions, and predator avoidance (Brodin et al., 2014; Cerveny et al., 2020; Painter et al., 2009; Schultz et al., 2011; Valenti et al., 2012), amongst others. These ecotoxicological effects have been demonstrated at environmentally relevant concentrations in surface waters (e.g., Schultz et al., 2011). Despite large data gaps still hindering proper risk assessment, where data are available, it is clear that the presence of psychopharmaceuticals can pose a risk to the aquatic ecosystems (Davey et al., 2022).

Liquid chromatography-high resolution mass spectrometry (LC-HRMS) techniques have improved over recent years, which has led to an increase in occurrence data for psychopharmaceuticals (Heberer, 2002; Luo et al., 2014; Ort et al., 2014; Richardson, 2007). Often it is antidepressants, antiepileptics, and painkillers that occur in the highest concentrations (Davey et al., 2022), with an extreme example of paracetamol, fluoxetine, and carbamazepine found in concentrations approaching or exceeding 1 mg/L in Almeria WWTP effluent in Spain (Bueno et al., 2012). High effluent concentrations have led to the occurrence of pharmaceuticals in surface waters around the world (Aus der Beek et al., 2016; Davey et al., 2022; Fonseca et al., 2020; Gago-Ferrero et al., 2020; Mheidli et al., 2022; Nozaki et al., 2023; Silva et al., 2011; Wilkinson et al., 2022). The prevalence of these compounds in effluent and surface waters has triggered scientists and institutions to monitor these compounds (Rijkswaterstaat, 2021; RIWA-Rijn, 2021) and to build databases of occurrence data (e.g., Eike et al., 2019; NORMAN Network, 2021).

At the same time, many highly prescribed or used compounds are still understudied, sometimes due to a lack of (affordable) analytical standards (Davey et al., 2022), or are widely unmonitored (e.g. Rijkswaterstaat, 2024, 2021; see online supplementary material SI-2). Even for the compounds that have been analyzed, many still lack measured WWTP removal data (Fawzi et al., 2021). In the coming decades, many Dutch WWTPs will undergo upgrades to improve water treatment (Spit et al., 2023) as is expected throughout Europe (van Dijk et al., 2023). The European Union’s proposal for an urban wastewater treatment directive (European Commission, 2022) proposes progressively more rigorous measures over time. It is vital to understand how well these upgrades can increase the removal of psychopharmaceuticals, yet discussions on how to measure removals remain ongoing.

One proposal for defining removal targets in the urban wastewater treatment directive is a risk-based approach determined by the member state (See Article 18, European Commission, 2022). By comparing the effluent concentration and the ecotoxicity of a compound, a conservative risk assessment can be performed to determine if a psychopharmaceutical might present an ecological risk. A risk can be due to a low removal efficiency, high prescription and use, high inherent toxicity, or a combination thereof. As such, the risk assessment approach could allow for a more granular look into removal efficiencies of micropollutants, which could be an advantage over flat removal targets (Bourgin et al., 2018; Stichting Toegepast Onderzoek Waterbeheer, 2020; Svahn and Borg, 2024) or population caps for treatment levels (European Commission, 2022).

Therefore, this study aimed to perform target screening on psychopharmaceutical occurrence in influent and effluent samples from the Amsterdam West WWTP during 1 week. Liquid chromatography-high resolution mass spectrometry is used to measure concentrations of psychopharmaceuticals from a preselected target list of compounds that was based on their risk in the literature or high prescription and use. Using the quantitative target data, we investigate the removal efficiencies of these compounds and contextualize the data by performing a preliminary risk assessment using existing ecotoxicity data. This pairing of ecotoxicity to removal was done to investigate the merits and drawbacks of such a method should it be used in legislation.

## Materials and methods

Supplementary information for this article can be found online in an Excel file containing 14 supplementary tables. Details on the tables can be found at the top of each sheet.

A new extraction method for all compounds was developed for this study. A full explanation and results of the method validation can be found in the method validation supplementary document and in section SI-8 of the online supplementary information Excel file. The method validation indicated that 30 of the 42 target compounds had sufficient recoveries and limited matrix effects and therefore were included in the study.

### Target compound selection

Psychopharmaceuticals, as defined in Davey et al. (2022), are approximately 700 compounds based on the ATC-N class of pharmaceuticals (World Health Organisation Collaborating Centre for Drug Statistics Methodology, 2018), and other psychoactive compounds (e.g., caffeine, illicit drugs). From the 700 potential compounds, this study focused on highly prescribed compounds based on Dutch prescription data (See online supplementary material SI-2; Zorginstituut Nederland, 2022), supplemented with commonly used compounds (e.g., caffeine, over-the-counter analgesics) and compounds that were understudied in the literature or had been shown to demonstrate a risk in surface waters (Davey et al., 2022). This resulted in a target list of 42 compounds (See online supplementary material SI-1), which was shortened to 30 after method validation (see Method validation, online supplementary material SI-5).

### Materials

Amsterdam West WWTP was used for the samples in this study. It was built between 2003 and 2005 and was connected to 767,000 population equivalents and local industry at time of sampling. The WWTP uses a primary sedimentation, activated sludge (modified University of Cape Town, mUCT) process, followed by a secondary sedimentation tank, and is attached to a combined sewage system (van Nieuwenhuijzen et al., 2009). A total of seven effluent and seven influent samples, taken by Waternet (Amsterdam water authorities) and supplied via KWR Water Research Institute, were analyzed. The samples were taken over seven consecutive days between March 18 and 24, 2021 (See online supplementary material SI-3). The samples were 24-hr composite samples and were collected using an autosampler that collected volume-proportional samples of 50 ml every 350 m^3^; this resulted in at least 300 subsamples per sample. The specific week was picked to be a representative “normal” week, because it fell outside holiday seasons and without specific events in the WWTP catchment area. A subsample of 250–300 ml of each sampling day, collected in HDPE bottles, was transported to the laboratory, where the samples were stored at –20 °C within 24 hr of sampling until extraction. Before analysis, the samples were put in 4 °C to defrost overnight and extracted the next day.

The labeled and unlabeled standards used for both the method optimization and measurements were purchased from Sigma-Aldrich Chemie (Schnelldorf, Germany; see online supplementary material SI-4). All unlabeled standards were analytical grade certified reference materials (≥ 98% purity). Stock solutions were prepared using MeOH and stored at –20 °C. Oasis HLB cartridges (6 cc, 150 mg) were purchased from Waters (Etten-Leur, the Netherlands). The solvents used for solid phase extraction (SPE), chromatographic separation, and stock solutions were of LC-MS grade from Biosolve (Valkenswaard, the Netherlands). Ultrapure water used for SPE, and separation was produced by a Milli-Q Direct Water Purification System from Merck (Damstadt, Germany).

### SPE

Effluent samples were spiked with 1 μg/L of labeled internal standards and shaken at 90 rpm for 30 minutes. Prior to SPE, outlets, tubes, and adapters were cleaned with 10 ml ultrapure water followed by 10 ml MeOH to prevent cross-contamination between SPE batches. The SPE method was based on Asghar et al. (2018). Conditioning of the Oasis HLB cartridge (150 mg, 6 cc) was done with 6 ml MeOH and 6 ml ultrapure water. After sample loading (50 ml, in duplicate), the cartridge was dried for 30 min under vacuum and washed with 6 ml ultrapure water. Before elution, a 0.22 µm polypropylene syringe filter was placed between the cartridge and SPE inlet. Elution was achieved with 2 × 5 ml MeOH under vacuum. The collected elution fractions were evaporated under a gentle nitrogen flow at 37 °C to <1 ml and reconstituted to 1 ml in MeOH. Elution fractions were diluted 5 times with ultrapure water prior to analysis to better match the mobile phase of the LC separation. A concentration factor of 10 was achieved with the SPE and evaporation method as described above.

For the influent samples, 5 ml influent was diluted with 45 ml ultrapure water, yielding 50 ml diluted influent. These diluted samples were then extracted in the same manner as described for the effluent samples. The influent samples achieved a concentration factor of only 1 due to the initial dilution.

### LC-HRMS method

Liquid chromatography of the samples was performed with an ultrahigh-performance LC system (Nexera 30 Schimadzu, Den Bosch, The Netherlands). The LC column used was an Acquity UPLC CSH C18 column (130 Å, 2.1 × 150 mm, 1.7 μm, Waters Corporation, Milford) kept at a temperature of 40 °C. The mobile phases consisted of ultrapure water (Milli-Q) with 0.05% acetic acid (A) and MeOH (B). The gradient started with a 7-min equilibration at 10% B and gradually increased to 100% B in 10 min, held at 100% B for 5 min, and back to 10% B in 0.5 min, totaling 22.5 minutes. The flow rate was 0.3 ml/min and the injection volume 20 µL. For compounds above the linear quantification range, a second round of injections was performed with an injection volume of 5 µL.

The LC was coupled to a maXis 4G quadrupole time-of-flight HRMS (qToF/HRMS) upgraded with HD collision cell and electrospray ionization (ESI) source (Bruker Daltonics, Leiderdorp, The Netherlands). Electrospray ionization+ and ESI- were achieved in individual runs by acquiring HRMS1 spectra for 20–1,000 *m/z* with a resolving power of 30,000–60,000 at full width half maximum with a spray voltage of +3.5 kV and –3.5 kV for positive and negative modes, respectively. The HRMS was calibrated prior to analysis of the samples and individual injections in both ESI+ and ESI- modes using a 2 mM sodium acetate solution in H_2_O: MeOH (1:1, v: v) direct injection. Per-sample internal calibration was achieved using a 50 µM sodium acetate solution in H_2_O: MeOH (1:1, v: v) infused with a 20 µl loop injection. Qualification of target compounds was based on the mass accuracy of full-scan HRMS spectra and tandem mass spectrometry (MS/MS) ions acquired in data-independent MS/MS mode, and their retention time match with the calibration series (See *Qualification and quantification method* section). In total, 32 psychopharmaceuticals passed the sensitivity and separation criteria (See online supplementary material SI-5); for these, SPE method validation was performed (see Method validation online supplementary document and online supplementary material SI-8).

### Qualification and quantification method

Qualification of psychopharmaceuticals was carried out with TASQ Ver. 2021.0 316 (Bruker Daltonics, Leiderdorp, the Netherlands) using broadband collision-induced dissociation (bbCID) MS/MS data to find precursor (quantifier) and fragment (qualifier) ions matching existing spectra in Massbank (Horai et al., 2010). Some compounds did not use qualifier ions in the method due to poor fragmentation, or because the analyte had matching fragments with its labelled internal standard which interfered with qualification (See online supplementary material SI-6).

During quantification, only the chromatograms with a retention time of >1 min, retention time tolerance of ± 0.3 min, mass tolerance of 0.002 Da, a detectable qualifier ion, mSigma of <100, and a peak intensity of >1,000 were considered. Target chromatograms were compared with calibration and Internal Standard (IS) chromatograms to check if the automated integration was successful and manually integrated if not. Calibration curves for quantification were calculated by analyzing ultrapure water spiked with target compounds and serially diluted to obtain 18 concentration levels for all target compounds (See online supplementary material SI-7). The 18 solutions were further spiked to contain 10 µg/L of IS. Concentration calibration was achieved using the calibration series in TASQ, with a minimum of seven levels with a residual of <30% and a linear *R^2^* of >0.99. The calibration range was optimized per compound per analysis to obtain the lowest feasible limit of detection (LOD) and the highest quantifiable concentration. Sample IS area was compared with the calibration IS area to determine the per-sample recoveries and adjust for losses. Amphetamine, bupropion, and phenacetin used ISs of other comparable but not identical compounds and are therefore considered as semiquantified (See online supplementary material SI-4). Further analyses were carried out in Microsoft Excel, where blank concentrations were subtracted, duplicates averaged, and dilution factors were accounted for to give final sample concentrations (See online supplementary material SI-11).

### Removal efficiency and risk analysis

Removal percentage is calculated per compound by taking the ratio of the effluent and influent on each of the days of sampling. Because there is a residence time of approximately 1 day in the Amsterdam West WWTP, the removal can be calculated for 6 days. If a compound was below the LOD or the limit of quantification (LOQ) in the effluent, the LOD or LOQ was used to determine the minimum removal efficiency for that compound.

To contextualize removals, a preliminary risk assessment was performed on the effluent data, using readily available ecotoxicity data. Freshwater predicted no-effect concentration (PNEC) values were taken from the NORMAN PNEC database (See online supplementary material SI-10, NORMAN Network, 2021). To show how risk can inform required removal efficiencies, risk quotients (RQs) were calculated per compound per day of effluent sampling using the measured effluent concentrations divided by the PNECs. From these data, risk boxplots were created showing the variation in risk over the 7 days. During this nonconservative preliminary risk analysis, no further assessment factors were applied, contrary to what is standardly done for deriving environmental quality standards. For compounds below the LOD or the LOQ in the effluent, the LOD or LOQ was used for the risk calculation.

With regard to the antidepressant citalopram, there are two stereoisomers in use in the Netherlands, citalopram and escitalopram. The analytical method used does not distinguish the isomers and, therefore, the combined concentrations for both isomers are reported. To correct for this in the correlations and risk analysis, the prescriptions for citalopram and escitalopram were added together, and the more conservative PNEC (escitalopram) was used for the risk analysis.

### Correlations and expected load

To test the reliability of the gathered data, Pearson correlations were run with log-transformed Dutch prescription data from 2021 for the 26 compounds that were only prescribed and not sold over the counter, taken from GIPDatabank (See online supplementary material SI-2,12, Zorginstituut Nederland, 2022). To adjust for human metabolism, excretion fractions were taken from DrugBank (Wishart et al., 2018) or the European Medicines Agency (See online supplementary material SI-13) if not available on DrugBank, and median values were used if a range was presented. The excretion fractions were multiplied with the number of defined daily doses (DDDs, i.e., prescription) and the DDD size (dose size; See online supplementary material SI-2, Norwegian Intitute of Public Health, 2024) to get the expected load (Equation [1]; See online supplementary material SI-12). This calculation was designed to test whether the gathered data is in line with the other literature (Davey et al., 2022; He et al., 2020; Oosterhuis et al., 2013; ter Laak et al., 2010).


(1) Expected Load=DDDs·EF·DDD size

## Results and discussion

### Wastewater occurrence

#### Influent occurrence

**Figure 1.**
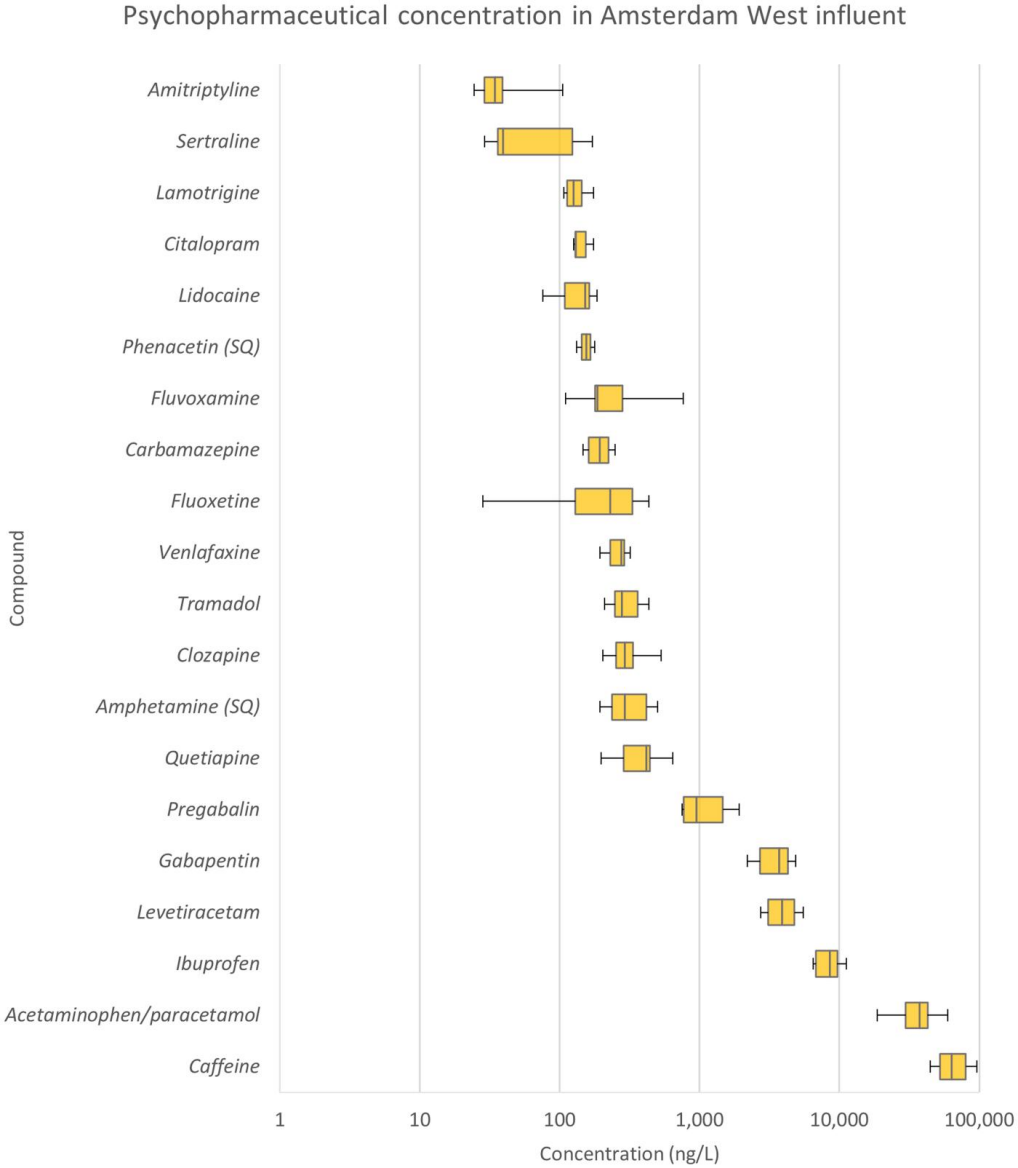
Concentrations of the 20 quantifiable psychopharmaceuticals in Amsterdam West wastewater treatment plant influent. The boxplots show the variation cross the 7 days tested (with exception for fluoxetine [2], phenacetin [2], and sertraline [5]), where the box and whiskers represent quartiles and the line inside the box represents the median.

In total, 20 out of the 30 measurable compounds were detected in Amsterdam West influent above their LOQs (Figure 1), whereas paroxetine could be detected but not quantified, and nine compounds could not be detected (below LOD, See online supplementary material SI-9). Concentrations of all quantifiable psychopharmaceuticals ranged from 24.6 ng/L to 96 µg/L (See online supplementary material SI-11). The relative SD for interday differences was 35% on average, including compounds with some sampling days <LOQ. Thus, the daily variation for psychopharmaceuticals quantified in influent is fairly low, which is to be expected with the prescription instructions and consumption habits for this type of pharmaceutical. Caffeine is mainly used recreationally, and amphetamine is known to be used illicitly and therefore may have had higher daily variations; however, the relative interday SDs for these two compounds fall in line with the other compounds (See online supplementary material SI-11).

Concentrations in the influent correlated with metabolism-adjusted prescription data (i.e., expected load, see online supplementary material SI-12) from the same year (*r*[25] = 0.63, *p* ≤ 0.05; see online supplementary material SI-12). Mianserin did not have excretion data and so could not be included in the correlation. These results are in line with previous literature on pharmaceuticals (Davey et al., 2022; He et al., 2020; Oosterhuis et al., 2013; ter Laak et al., 2010) and are indicative of the reliability of the data gathered. The three compounds with the highest concentrations were paracetamol (acetaminophen), caffeine, and ibuprofen, which are over-the-counter analgesics (paracetamol and ibuprofen) and a legal recreational stimulant. Because of how these compounds are consumed, these are not included in the correlation (See online supplementary material SI-13).

#### Effluent occurrence

**Figure 2.**
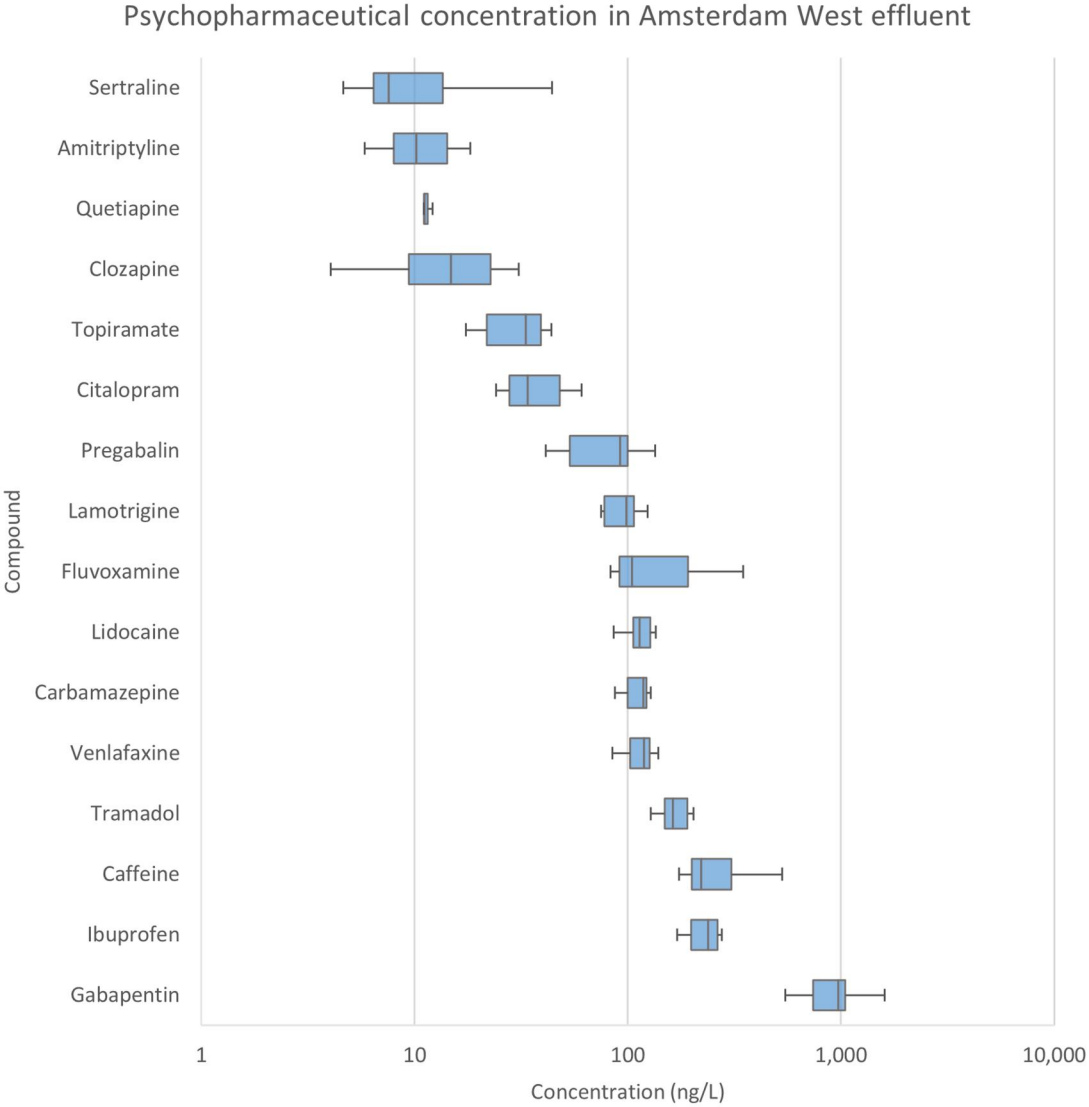
Concentrations of the 16 quantifiable psychopharmaceuticals detected above the limits of quantification in Amsterdam West wastewater treatment plant effluent. The boxplots show the variation cross the 7 days tested (with exception for pregabalin (6), topiramate (6), citalopram (6), quetiapine (5), clozapine (3), and amitriptyline (3)).

Of the 30 measurable compounds, 16 were detected in the Amsterdam West effluent above their LOQs (Figure 2), with eight compounds being below the LOD and six compounds detected but unquantifiable (See online supplementary material SI-9). Concentrations in effluent ranged from 4 ng/L to 1.6 µg/L (See online supplementary material SI-11). The relative SDs for interday differences were 34% on average. Compounds with some days reporting concentrations <LOQ have higher relative SDs. Rainfall might affect concentrations in the combined sewer system; however, the weather in Amsterdam during the period of sampling was quite consistent (overcast, no precipitation, and daytime highs of 8–11°C; Royal Netherlands Meteorlogical Institute, 2023).

Effluent concentrations did not correlate with prescription data from the same year ( *r*[25] = 0.26, *p *=* *0.21; see online supplementary material SI-12), as was expected, due to different fate of the chemicals within the WWTP (see *Removal efficiencies and preliminary risk analysis* section). Like the influent, highly used and over-the-counter compounds such as ibuprofen and caffeine showed high effluent concentrations, with the notable exception of paracetamol, which was below the LOD in effluent (See online supplementary material SI-9). Gabapentin showed the highest median concentrations, explained by the high expected load (See online supplementary material SI-12) in combination with limited WWTP removal efficiency (see *Removal efficiencies and preliminary risk analysis* section).

### Removal efficiencies and preliminary risk analysis

**Figure 3.**
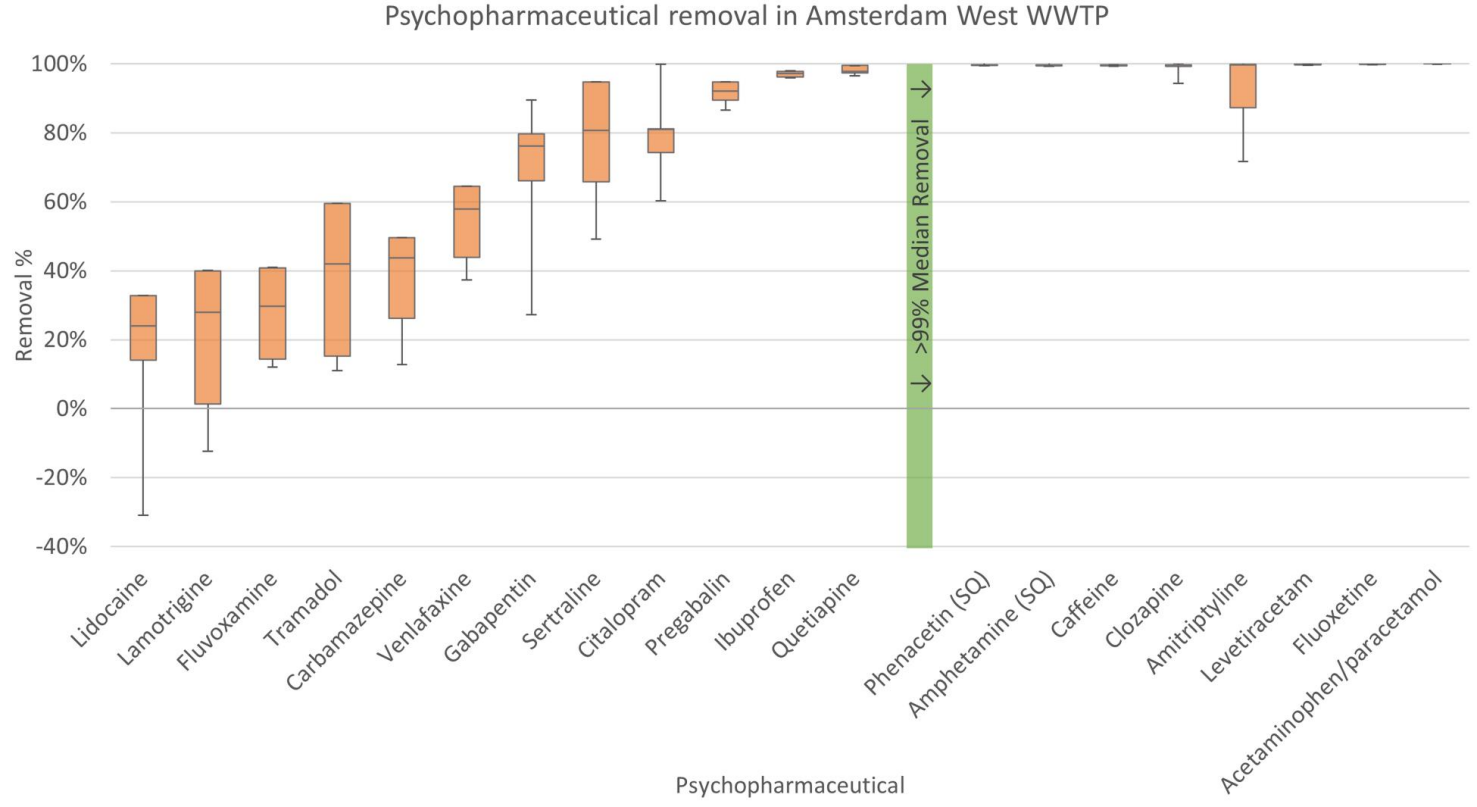
Removal efficiencies (concentration ratio of effluent over influent, expressed as percentages) of the psychopharmaceuticals found in Amsterdam West wastewater treatment plant (WWTP) ordered by increasing median removal. The boxplots show the variation in removal across the days tested where the box and whiskers represent quartiles and the line inside the box represents the median concentration.

A total of 20 compounds had sufficient data for the removal efficiency to be calculated. All these compounds showed positive removal, except for lidocaine and lamotrigine, which showed a negative removal (and thus a higher concentration in the effluent compared with influent) on 1 to 2 days (Figure 3 and online supplementary material SI-11). Eight compounds (phenacetin, amphetamine, caffeine, clozapine, amitriptyline, levetiracetam, fluoxetine, and paracetamol) returned > 99% removals.

**Figure 4.**
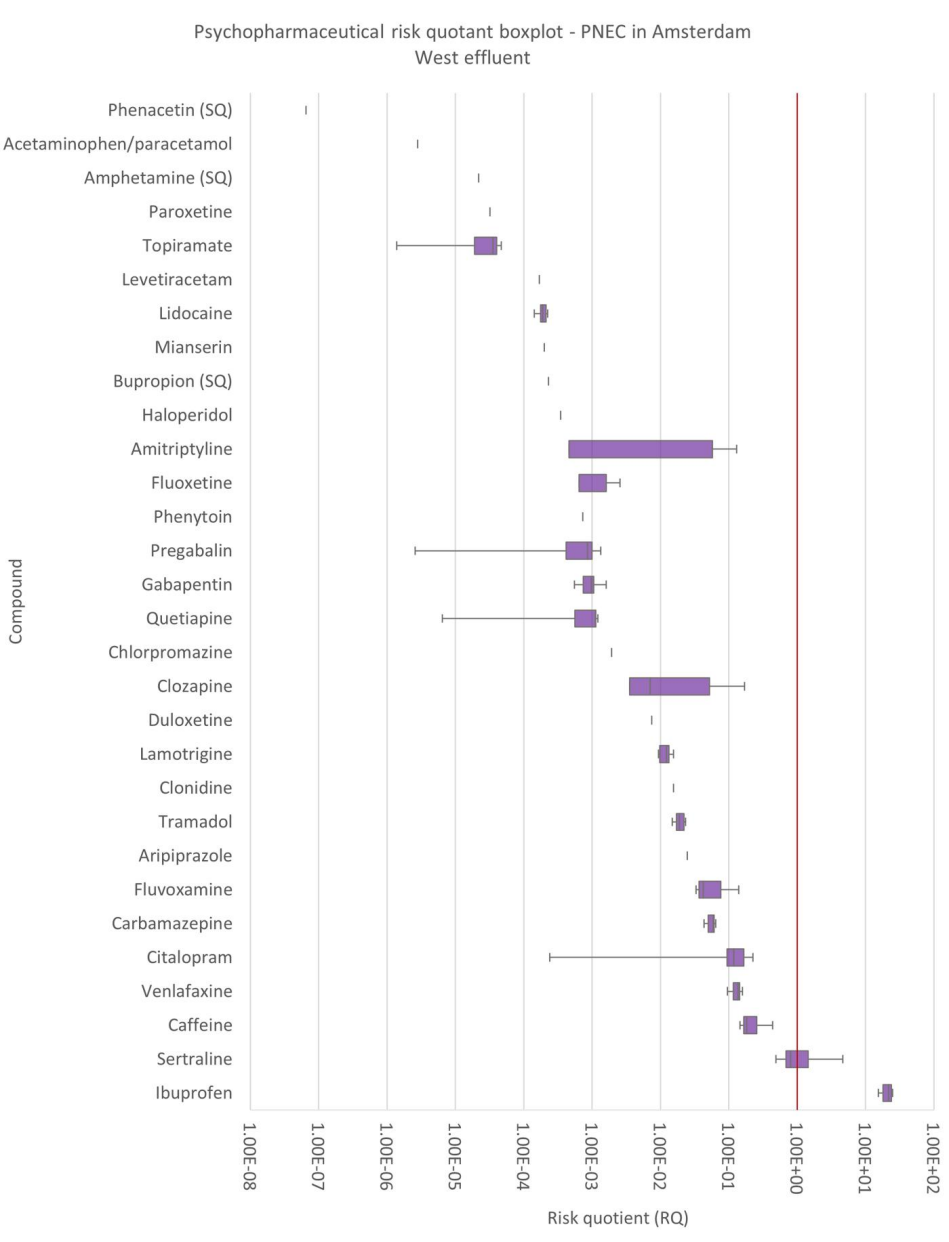
Risk quotient (RQ) boxplots for the 16 psychopharmaceuticals found in Amsterdam West wastewater treatment plant effluent, and the 14 compounds below the limit of detection/limit of quantification represented by dashes, ordered by increasing median risk. The boxplots show the variation across the days, where the box and whiskers represent quartiles, and the line inside the box represents the median risk. A risk quotient (RQ) of 1 (without further assessment factors) has been highlighted by the verticle line.

A total of 16 compounds could be quantified in the effluent and could therefore be compared with PNECs to perform a preliminary risk assessment. There were 14 compounds below the LOD or LOQ, but they could still be compared with PNECs using these parameters. Sertraline and ibuprofen show a definitive risk (Figure 4), whereas clozapine, fluvoxamine, amitriptyline, caffeine, venlafaxine, and citalopram show at least 1 day less than one order of magnitude away from demonstrating a risk (See online supplementary material SI-10).

## General discussion

### Compatibility with the literature

When comparing the concentrations found with databases in the literature, this study generally reported significantly lower effluent concentrations, even when filtering only for the Netherlands (Eike et al., 2019; NORMAN Network, 2021; Rijkswaterstaat, 2021; see online supplementary material SI-14). Furthermore, although all 30 compounds searched for do appear in the European databases, five compounds (aripiprazole, phenacetin, phenytoin, pregabalin, and topiramate) have not been included in monitoring data or studies in the Netherlands, to the best of our knowledge. This remains true when accounting for surface water monitoring (Rijkswaterstaat, 2021; see online supplementary material Table SI-13). Given the differences in factors such as individual WWTP removal and national prescription regimes, it is important to have local data when assessing risk; thus, this study helps to fill some of the local data gaps for the Netherlands.

### Removal–risk pairing and PNEC robustness

This study pairs WWTP removal efficiency to ecotoxicity in a preliminary risk assessment of the effluent concentration to contextualize removal efficiencies. Specifically, we show that compounds that meet general chemical removal targets (e.g., 70%, Stichting Toegepast Onderzoek Waterbeheer, 2020, or 80%, Bourgin et al., 2018; Svahn and Borg, 2024) can still have a risk (e.g., sertraline and ibuprofen, Figure 4) whereas other compounds that do not meet the targets do not pose a risk. The approach further allows the deduction of the cause of these risks. For example, sertraline has the lowest PNEC, which produced the highest RQs (see online supplementary material Table SI-10), indicating that it is the high ecotoxicity of this compound that contributes most to the risk. In the case of ibuprofen, it is a combination of its moderate ecotoxicity and high load that produces the risk (see online supplementary material Table SI-10, Figure 1).

The preliminary risk analysis used the lowest freshwater PNECs taken from the NORMAN Network (NORMAN, 2021), aiming to keep the risk assessment simple using readily available data. These PNECs contain a mixture of PNECs derived from both experimental data and modeled data and use different assessment factors (See online supplementary material SI-10; this study did not add any additional assessment factors). The NORMAN database allows for details per entry (yet often these are missing), such as whether acute or chronic data was used or what the exact endpoints were. Missing ecotoxicity data is known to be a major limitation in risk assessment for psychopharmaceuticals (Davey et al., 2022), and this extends to the NORMAN PNECs. By coupling removal to ecotoxicity better insights can be gained than looking solely at removal (see *Removal and risks of comparable psychopharmaceuticals* section). However, more extensive ecotoxicity data and robust (chronic) PNECs could provide more accurate risk assessments, as would behavioral endpoints, which can be more sensitive than nonbehavioral endpoints and are of specific interest for psychopharmaceuticals (Davey et al., 2022; Raman et al., 2024; Schuijt et al., 2023).

### Removal and risks of comparable psychopharmaceuticals

Despite the high removal efficiency of ibuprofen, it still demonstrates the highest risk of all quantifiable compounds in this study with an RQ range of 15–25 (See online supplementary material SI-10), presumably due to its high use volumes and moderate ecotoxicity (See online supplementary material SI-9,10). The risk derived from ibuprofen is a result of the approximately 3% of parent compound not removed (See online supplementary material SI-11). Although ibuprofen is also used as an anti-inflammatory drug, both paracetamol and ibuprofen can be used as over-the-counter analgesics or painkillers. Both drugs can be used for mild treatment of similar pain-related conditions, and for many people, can be used interchangeably (Drugs.com, 2023a, 2023b). Phenacetin, a compound related to paracetamol, showed a very high removal and no risk. The results suggest that the use of aniline-derived analgesics (ATC N02BE, World Health Organisation Collaborating Centre for Drug Statistics Methodology World Health Organisation, 2018) such as paracetamol and phenacetin instead of ibuprofen, when used as an analgesic, could reduce potential ecological risks. Considering paracetamol and ibuprofen are both over-the-counter drugs, such a substitution could be stimulated via public awareness campaigns. This is in line with suggestions by the Organisation for Economic Co-operation and Development (OECD) for an awareness campaign via ecolabeling for over-the-counter products (OECD, 2019).

The selective serotonin reuptake inhibitor antidepressants citalopram and sertraline potentially demonstrate a similar case, despite the limitations mentioned in the *Removal–risk pairing and PNEC robustness* section. Citalopram (including escitalopram) showed a removal efficiency of 60%–100%, whereas sertraline showed a range between 49% and 97%. Sertraline demonstrated a potential risk whereas citalopram did not (Figure 4), even when using the more conservative PNEC for escitalopram. This is despite the fact that sertraline had the lowest concentration of all compounds quantified in the effluent (Figure 1), indicating that its risk comes from the high ecotoxicity of the compound (See online supplementary material SI-10).

Additionally, (es)citalopram and sertraline had similar amounts consumed in 2021 (2,000 and 2,300 kg, respectively; see online supplementary material SI-2) despite citalopram being the most prescribed psychopharmaceutical in the Netherlands in terms of DDDs, approximately double the amount for sertraline. The smaller difference in loads comes from the fact that the DDD size for sertraline is 50 mg whereas that of citalopram is 20 mg and escitalopram is 10 mg. The lower dose sizes indicates that citalopram, and especially escitalopram, are far more efficient treatments in terms of chemical used.

The recommendations by the OECD (OECD, 2019) indicate a number of use-orientated options to reduce pharmaceutical residues. Because psychopharmaceuticals are often used to treat chronic mental health and nervous conditions, some of these options are either not applicable or difficult to achieve (e.g., reduction in incidence or reduction in self-prescription). Yet the comparison between (es)citalopram and sertraline this gives credence to some of the OECD suggestions, namely the optimization of dosing when it can be assured that the quality of treatment to the patient remains the same.

### Transformation products

This study did not quantify transformation products, that is, human metabolites and transformation products formed in the sewer system, WWTP, or environment, because this would require (labeled) analytical standards of a spectrum of potential metabolites, which are not easily available. There are legitimate reasons for studying transformation products, especially because these compounds may be found in higher concentrations than their parent compounds and may be biologically active and thus pose a risk (de Jongh et al., 2012; ter Laak et al., 2014; Zhi et al., 2021; Maculewicz et al., 2022). Using nontarget/suspect screening (e.g., Helmus et al., 2022) to identify transformation products, followed by (semi-)quantification to identify potential risky compounds, and only then followed by full quantification, could be a potential risk-driven approach.

### Urban wastewater treatment directive proposal and advanced treatments

The European Union’s proposal for an urban wastewater treatment directive (European Commission, 2022) proposes progressively more rigorous measures over time, starting from 2025 for WWTPs connected to populations of more than 100,000 to include advanced treatment technologies. There are numerous options for advanced treatments (Angeles et al., 2019), and the proposal places the selection of the treatment type on the member state, including selection criteria, with some suggestions. A risk-based approach (Article 18), such as the one presented here, is presented as one such suggestion. The results from this study clearly show the benefits of this risk-based approach, such as establishing the cause of the risk (high loads, high toxicity, etc.). Coupling toxicity with removal could be advantageous in fine-tuning removals when resources are limited; however, concerns relating to ecotoxicity data would need to be addressed for these types of risk assessment to be considered robust.

## Conclusion

This study developed an analytical method to quantify the occurrence of psychopharmaceuticals in Amsterdam West WWTP influent and effluent to estimate WWTP removal efficiency and conduct a preliminary risk analysis based on effluent concentrations to inform removal targets.

Although some compounds showed > 99% median removal, others varied between 24% and 98%. This shows that modern conventional WWTPs are currently not capable of effectively removing many psychopharmaceuticals, and two compounds (ibuprofen and sertraline) even demonstrated an ecological risk despite high removal.

Furthermore, risks may be assessed as more severe when factoring in missing ecotoxicological data, such as sensitive behavioral endpoints, or even the lack of experimental data. In combination, these missing data may lead to even higher ecological risk than those estimated in this study. Therefore, it is important to investigate options for reducing the ecological risk of psychopharmaceuticals, such as intraclass substitutions and advanced WWTP treatments. With further study, it may be possible to integrate risk assessment into removal targets in policy to come to a toxic free environment.

## Supplementary Material

vgae042_Supplementary_Data

## Data Availability

Calculated data are available in the supplementary information. Other data, such as LCMS data (both filtered and raw), can be made accessible on request from the authors.
