# Supplementary material for: Presence, removal, and risks of psychopharmaceuticals in wastewater streams
Source: Environ Toxicol Chem. 2025 Jan 6;44(2):375–85. doi: 10.1093/etojnl/vgae042 (PMC11816319; doi:10.1093/etojnl/vgae042)
Supplement: vgae042_Supplementary_Data [file vgae042_supplementary_data.zip › vgae042_Supplementary_Data/Method Validation Supplimentary.docx]

**Method Validation**

*MV-1 Introduction*

Recoveries and matrix effects needed to be established for all compounds in this study. By having recoveries and matrix effects between 60-140% could a method be considered validated, and therefore included in this study. This needed to be performed in both effluent and influent matrices.

10 compounds did not work on either the LC or the MS, and were dropped from the study prior to the SPE method validation (See SI-5 for details). This meant that 32 compounds were tested for the SPE method validation, with two compounds failing the validation and were therefore dropped from the study (see the results section).

*MV-2 Methods*

Method validation for effluent samples was performed on grab samples from Rhenen RWZI (The Netherlands) which is connected to 46,000 population equivalents and consists of a primary sedimentation tank, aeration tank, and a secondary sedimentation tank. We used a different WWTP for the method validation as we did not have enough Amsterdam effluent to test different methods on. Influent used for method validation was a non-proportional mixture of 6 different Dutch WWTP influents; Amsterdam, Rotterdam, Rotterdam Dokhaven, Utrecht, Eindhoven, and Zwolle. This was done because there was not enough Amsterdam influent to perform method validation.

*MV-2.1 Effluent Method Validation*

16 samples and two blanks were extracted using the same methods described in 2.3 with the exception of the addition of spiked standards. Spiked standards were added as shown in Table MV-1. Briefly, the *validation samples* followed the same protocol as described in 2.3 (1 µg/l labelled standard added before SPE), *pre-spiked sample* added 1 µg/L of labelled and unlabelled before SPE, *post-spiked sample* added 10 µg/L of labelled and unlabelled into the vial before injection, and *matrix effect sample* added 10 µg/L of labelled into the vial before injection. Any compounds that did not pass the effluent method validation were not tested in the influent method validation.

| Sample Type | Replicates | Standards Added before SPE (1 µg/l) | Standards Added before LCMS (10 µg/l) |
| --- | --- | --- | --- |
| Validation Sample | 4 | Labelled | - |
| Pre-Spiked Sample | 4 | Labelled, Unlabelled | - |
| Post-Spiked Sample | 4 | - | Labelled, Unlabelled |
| Matrix Effect Sample | 4 | - | Labelled |
| Blank | 2 | Labelled | - |
|  |  |  |  |

*Table MV-1: Overview of SPE method validation samples*

The concentration of the clean matrix was determined by making two vials containing 10 µg/L of labelled and unlabelled standards in ultrapure water. Some studies use the calibration vials for this, but 10 ug was not part of our calibration series, so we opted simply to make a new vial. The Blank SPE samples in Table MV-1 were used for blank subtractions as described in 2.5. The recovery for each compound was calculated using Equation MV-1, while matrix effects were calculated using Equation MV-2. Only recoveries and matrix effects of between 60-140% were considered to be acceptable. Compounds failing this criterium were excluded from analysis. Limits of detection were considered as the concentration of the lowest calibration concentration with a peak of signal to noise ratio of >3, while limits of quantification were considered the lowest calibration concentration within linearity (SI-8, SI-9).

$RE\%=\frac{C_{pre-spiked}-C_{validation}}{C_{clean matrix}}\cdot100\%$ $ME\%=\frac{C_{post-spiked}-C_{matrix effect}}{C_{clean matrix}} \cdot100\%$
 *Equation MV-1: Recovery (RE) calculation* *Equation MV-2: Matrix effects (ME) calculation*

*MV-2.2 Influent Method Validation*

Any compounds that did not pass the effluent method validation were not tested in the influent method validation. The influent method validation was the same as described in 2.1 of this document, but used the influent SPE method described in 2.3. In short, the influent was diluted 10x before the spiked standards were added, and was run through the LCMS twice with injection volumes of 20 ul and 5 ul, since some compounds may be overloaded. The results of the 20ul run was used by default, unless the peaks were overloaded, then the 5 ul run was used for those compounds, both in the method validation and in the main results.

*MV-3 Results*

Of the list of target compounds, 30 compounds could be detected with the developed LCMS method (Sections 2.4 - 2.6, SI-5) and showed acceptable recoveries and matrix effects in both influent and effluent (Figure MV1). Paracetamol and caffeine required the lower injection volume method for both influent method validation and influent quantification. Further details for all compounds tested, including LODs and LOQs, are provided in SI-5 and SI-8.

*Figure MV1: Recoveries (RE) and matrix effects (ME) for effluent and influent. Red lines indicate the boundaries of upper and lower acceptable recoveries and matrix effects. Compounds marked with ‘SQ’ are semi-quantified. Paracetamol and caffeine used the 5* $\mu$*L injection volume method for influent due to high background levels (details in SI-5 & SI-8).*
